# What is *Fragilaria koensabbei* and does it matter what it is called? (Meta)barcoding and the science of taxonomy

**DOI:** 10.1111/jpy.70020

**Published:** 2025-05-03

**Authors:** David M. Williams

**Affiliations:** ^1^ The Natural History Museum London UK

**Keywords:** diatom, molecular, taxonomy

## Abstract

Commentary is provided on the use of barcoding and metabarcoding in diatom studies and its broader relevance to the principles of taxonomy. The claims that taxonomy, however it is performed, is *time‐consuming* and that it requires *extensive expertise*, due to a *constantly evolving taxonomy*, are questioned as good, even useful, criteria by which to judge a science.

**Abbreviations**
ASVs: amplicon sequence variants
BSC: biological species concept
WFD: Water Framework Directive

Men fight and lose the battle, and the thing that they fought for comes about in spite of their defeat, and when it comes turns out not to be what they meant, and other men have to fight for what they meant under another name

(William Morris, *London Review of Books*, February 8, 2024. [From William Morris (1888) *A Dream of John Ball*])

II faloit chercher dans la nature elle‐méme son Systeme, s'il etoit vrai qu'ele en eut en.

(Adanson, 1763, p. clvii, quoted in Nelson, 1979).

*Fragilaria koensabbei* Al‐Handal and Al‐Shaheen (2019, p. 22, pl. 6, figures 1–9, pl. 4, figures 1–10) has its type locality in “Shatt Al‐Arab River and Huwiza marsh,” Iraq (Al‐Handal & Al‐Shaheen, 2019, p. 22; for a map of the area, see Al‐Handal & Al‐Shaheen, 2019, p. 5, figure 1 and Al‐Saedy et al., 2020, p. 240, figure 1). It has not been recorded often since its first description. Al‐Saedy et al. (2020) summarized its distribution as “Fresh to brackish water. Planktonic, Epiphytic [sic], epipelic, epilithic, NSH, MSH, SSH” with the abbreviations of “NSH, northern parts of Shatt Al‐Arab river, representing the region from Garma and upstream …, MSH, middle part of the river extending from Ashar city to Abu Flos region, and SSH, representing the southern part which extend from Siba to Faw towns” (pp. 246, 240). Given its known restricted distribution, it is likely to be endemic and, therefore, may be of some biogeographic significance (Williams, 2024).

In another contribution dealing with diatoms from the “Rivers Tigris and Euphrates in northern Basra, Southern Iraq,” Mohamad et al. (2024) used “morphological and metabarcoding analyses” in an attempt to speed up species identification: “The microscopic‐morphological analysis of the environmental samples resulted in the identification of 284 infrageneric taxa in 59 genera. For the metabarcoding analysis the V4 region of the 18S marker gene was used and resulted in 1454 ASVs (Amplicon sequence variants) comprising 54 genera and 108 species with several ASVs belonging to the same taxa” (p. 411 [abstract]). They included 11 “figures” (plates) of light micrographs of diatom valves from numerous species; in most cases, there was one image per species (their figures 2–11; species names and their image in the figures are listed in table 3 of Mohamad et al., 2024). One of the images is named *Fragilaria koensabbei* (Mohamad et al., 2024, figure 3G, the figure is labeled “LM pictures of taxa found by morphological analyses”; see also Mohamad et al., 2025, p. 65, table 2, without figure).

Without the need to collect more data and without having to inspect any further images other than those published in Al‐Handal and Al‐Shaheen (2019) and Mohamad et al. (2024), it is clear that these specimens are not representative of a species of *Fragilaria* Lyngb. From the excellent published images of *Fragilaria koensabbei*, one might conclude otherwise: that they are better placed in *Tabularia*. Indeed, the authors compared their specimens only to species in *Tabularia* and did not mention other species of *Fragilaria* in their discussion (Al‐Handal & Al‐Shaheen, 2019, p. 22). Why so? This is discussed below, but first the species requires redescription:

**Tabularia koensabbei (Al‐Handal & Al‐Shaheen) D.M.Williams, comb. nov**.

Basionym: *Fragilaria koensabbei* (Al‐Handal & Al‐Shaheen, 2019, p. 22, pl. 6, figures 1–9, pl. 4, figures 1–10).

Registration: http://phycobank.org/105503.

*Revised description*: Frustules rectangular, elongated, free living, or attached at poles. Cells with several plate‐like chloroplasts. Valves linear to slightly lanceolate with parallel margins and flattened rounded poles. Valve face flat; margin at right angles between valve face and mantle. Virgae prominent, merging with broad sternum; vimines short “cross‐bars” extending across pairs of virgae. Closing plate absent. Ocellulimbus at both poles, slightly convex, composed of even rows of porelli. Rimoportulae at each pole embedded in sternum, opening externally as simple slit, internally as paired lips. Cingulum composed of four to five open bands, each with row of poroids, when in situ, situated just below valve margin; valvocopulae with crenulated edges to affix to valve.

This description differs in some of the details. For example, the structure of the valve (the “basal siliceous layer,” Ross et al., 1979, p. 525) is better captured by documenting their component parts (the *virgae* and *vimines*, Cox & Ross, 1981; the *sternum*, Round et al., 1990, etc.) rather than just noting the “stria,” which is not really a character but a composite. Additionally, and perhaps more importantly, the girdle is composed of open rather than closed bands, with a distinctive patterning along its midpoint (*pars media*). After redescription, it is clear this is a species of *Tabularia* or, more accurately, one of the subsets of that genus (Williams & Round, 1986, 1988). It may even be a synonym of *Tabularia sinensis* Yue Cao et al. (Cao et al., 2018, p. 180, figures 1–39), a proposition that requires investigation.

With respect to diatom identification, consider the following statement in Mohamad et al. (2024): “Accurate taxonomic identification of diatoms is essential for the sound evaluation of their diversity. However, diatom identification based on *counting and identifying their valves* using mostly light microscopy *requires highly specialized taxonomic skills* (Bíró et al., 2022; Stuart et al., 2024) and may lead to discrepancies in the findings depending on the researcher (Kang et al., 2021)” (Mohamad et al., 2024, p. 412, my italics). Many others apparently feel the same: “[…] the current methodology, based on morphological taxonomic identification, is *time‐consuming* (counting of 400 valves per sample under microscope), and requires *extensive expertise*, due to a *constantly evolving taxonomy*” (Bailet et al., 2019, p. 22, my italics); “*It requires time and trained analysts* with good knowledge of the taxonomic literature, but inter‐analyst variation can often influence the final result. In addition, many semi‐cryptic species and species complexes […] make morphological identification complex” (Kochoska et al., 2023, p. 1). Mohamad et al. add: “Furthermore, in regions where literature on the local diatom flora is not available, researchers often rely on European floras, which may force identifications into the existing taxonomic concepts, potentially overlooking local variation or unique species” (p. 412). Yet, *Fragilaria* is not the most obvious genus for the specimens named as *Fragilaria koensabbei*.

It might seem unusual, to say the least, to dismiss a science because it “requires time and trained analysts.” However, it does beg a question—does it matter what the specimens are called? After all, if one has a barcode or something similar, just search for that—the taxonomic name is surely irrelevant, and anyway, the “constantly evolving taxonomy” might cause yet another change. Again, this might seem an unusual way to dismiss an entire scientific discipline just because its conclusions (its hypotheses) might change with time as *relevant* evidence accumulates or as evidence already acquired is reevaluated (let me once again stress: *relevant* evidence). Yet, some pursue this to its logical (dead) end, with “taxonomy‐free diatom‐biomonitoring, in which environmental DNA metabarcoding data is utilised but not assigned to specific taxonomic classes, presents an accurate, fast, and relatively automated alternative to taxonomically assigned eutrophication biomonitoring” (Gregersen et al., 2023, abstract; see also Apothéloz‐Perret‐Gentil et al., 2017). Some make even more grandiose claims: “DNA (meta) barcoding shows particular promise thanks to its ability to identify organisms *objectively, affordably*, and (once the reference barcode databases are sufficiently developed) *without a need for specialized knowledge of the local floras*” (Kollár et al., 2025 p. 2, my italics); “We note that DNA metabarcoding can *largely eliminate inconsistencies in identification*, allow many more samples to be processed, and generate vast numbers of DNA reads” (Kulichová & Trobajo, 2024, abstract, again, my italics); and “Reliable identification of species from frustule morphology *requires a high level of skill*, *a thorough knowledge of the relevant literature*, and is *time‐consuming and expensive*” (Kulichová & Trobajo, 2024, p. 125, also my italics)—as if cost is a scientific criterion and knowledge a handicap (“a high level of skill … a thorough knowledge”), with objectivity being misconceived as a parameter of data, as if data come readily explained (Kollár et al., 2025, confuses *repeatability* with objectivity, but see Meier et al., 2022, for one of many criticisms of objectivity in barcoding; for a reasonable discussion on objectivity, see Suárez‐Díaz & Anaya‐Muñoz, 2008), with pipelines and protocols replacing intellectual coherence (Vasselon et al., 2025); and just think what could be achieved if there were 41 more diatom taxonomists (Engel et al., 2021).

Yet, what does the barcode actually point to? What biological entity does it imply? Anything? Nothing? Something? Does comparative biology (= systematics = taxonomy, see Williams & Ebach, 2020; Williams & Gill, 2025) matter anymore? Perhaps, the ecological community could do with a little more steeping in the subject. Consider the following: It is quite likely that the genus *Fragilaria* is *paraphyletic* as currently circumscribed—if not paraphyletic, then *aphyletic* (Ebach & Williams, 2010; Williams & Ebach, 2020). Why is this an issue? Although the word *paraphyly* gets used here and there in diatom systematics, is there some general understanding of its meaning? Of this, I am not sure. In terms of evidence (that is, once again, *relevant* data), it means that the genus (taxon) has no unique characters of its own, making it virtually impossible to assign anything to it except by fiat (Williams & Ebach, 2020)—it does not represent any actual entity, biological or otherwise: they are “timeless abstractions” (Brundin, 1972, p. 111, also Patterson, 1978, and many others), created not discovered, and not part of the world we all actually live in (“paraphyly exists only in an abstract world,” Nelson, 2000, p. 46). In terms of evidential support, the same applies to aphyletic taxa (Ebach & Williams, 2010; Williams & Ebach, 2020). One might consistently recover the same barcode, but what exactly is being recovered?

Contrary to the above, some molecular evidence appears to indicate that a few species named in the genus *Fragilaria* might constitute a monophyletic genus (Kahlert et al., 2019, but compare this with Zakharova et al., 2023 and see Williams, 2024 on ubiquitous names)—but this approach lacks any indication of what characterizes the genus: what its synapomorphies are, never mind what the included species should be. Adding a list of characters to the node of a “phylogenetic” tree is neither sufficient nor appropriate, as these are not necessarily *relevant* data—or even well‐thought‐out characters—as the list is usually just a mix of synapomorphies that apply to various unspecified higher ranks.

Somewhat ironically, as pointed out above, *Tabularia* is almost certainly paraphyletic as currently circumscribed (a proposition known since 1986, see Williams & Round, 1986, 1988, p. 313, and, more recently, Cao et al., 2018, p. 180), with the same proviso: As currently understood, it has no unique characters of its own. Why, then, place *Fragilaria koensabbei* in *Tabularia*, another nonmonophyletic group? Simply as a pragmatic solution, as these specimens have the characters of the larger group that includes a number of other genera (Williams & Van de Vijver, 2023); the characters known so far address a rank higher than genus. One might pause here: Specimens were initially named *Fragilaria koensabbei*, which is not a member of that genus; *Tabularia* is more appropriate as the basal siliceous layer of the valves has prominent virgae, merging with a broad sternum, and vimines as short “cross‐bars” extending across pairs of virgae, a structure seen in a number of other genera but all part of the same monophyletic group (Williams & Van de Vijver, 2023), and the specimens may be *Tabularia sinensis*. In other words, a problematic taxon—so how might one identify something that is currently difficult to characterize? To investigate the matter does take time.

Still, what might one offer to those who find taxonomy *time‐consuming*, who say that it requires *extensive expertise* and that there is a *constantly evolving taxonomy*? I defer to an earlier, albeit nondiatom, comment, directed at “discordance” but still appropriate in this context:

To some persons […] ultimate reality is chaotic. These persons tend not to become scientists, but if they do, they tend to become frustrated. […] The problem is to see order in apparent chaos through *critical observation and hypothesis testing*. Some of these persons become scientists, and systematists (Nelson & Platnick, 1981, pp. 31–2, my italics)

“They tend to become frustrated.” One senses the frustration in this following passage and—given its tone—a fair amount of discouragement:

Correct identification can depend on very subtle differences, such as whether the density of striation on the valves is 25 or 30 in 10 μm, tiny inflexions of the valve outline, or the occurrence and distribution of special pores or spines. To assess such characteristics and determine species accurately requires initial training in the basics of diatom biology and biodiversity, a high quality light microscope handled expertly, good powers of observation, access to scanning electron microscopy to check light microscope detail and examine structures beyond the resolution of the light microscope, an extensive library (because descriptions of species are scattered across many journals and books, some old and rare), and the advice of colleagues (because probably no diatomist since the 19th century, even Friedrich Hustedt, has had a comprehensive knowledge of all genera). (Mann et al., 2010, p. 559)

Yet, most of the items listed here are techniques to *generate* or *gather* data, with little effort at interpretation other than, once again, to defer to one or another flora. Yet, no one writing on this so‐called “morphological method” appears to include instructions on how to deal with the morphological data once acquired (apart from saying how to measure something or other—and how tedious it all is); there is no guidance as to what might be required to understand the *principles* of taxonomy, as if the act of species identification is merely the ability to gather data, read and interpret one or another flora, and tolerate the idiosyncrasies of the various taxonomists who have actually studied the organisms in question. One could avoid it altogether: “It might be possible, for example, to design a barcode system that largely emulates the widely used freshwater flora of Krammer & Lange‐Bertalot (1986–91), replacing microscopical features by molecular ones” (Mann et al., 2010, p. 568).

To their credit, the first volume of Krammer and Lange‐Bertalot's *Süßwasserflora von Mitteleuropa* does have a theoretical section, although it is limited to a justification of the species concept they used at that time, largely derived from an interpretation of the biological species concept (BSC) coupled with the workings of the then current Code of Botanical Nomenclature, as if the only rank worthy of consideration is the species (Krammer & Lange‐Bertalot, 1986, p. 74, “Systematiches konzept dieser Diatomeen‐Flora” [“Systematic concept of this diatom flora”]; a modified translation into English of this passage was included in Vol. 5, Krammer & Lange‐Bertalot, 2000a, p. 52 et seq.). The general systematics book they refer to in this section is one written by Ernst Mayr, the “creator” of the BSC (Mayr, 1975; this is the German edition/translation of Mayr, 1969—a number of other papers and books by Mayr are included in the reference list by Krammer & Lange‐Bertalot but never cited). In the “Vorwort” to the third volume of the *Süßwasserflora von Mitteleuropa*, reference is made to Simpson (1961, “Principles of Animal Taxonomy,” a largely paleontological perspective on taxonomy) and Sneath and Sokal (1973, their second summary of progress in “numerical taxonomy”). The bibliography, published in the fourth volume, oddly, includes reference to Hennig (1966, 1982, which is a detailed account of his “phylogenetic systematics” [or, to some, cladistics], the English [1966] and German [1982] editions, that were much discussed in the literature during the past half century, see Williams et al., 2016), along with a number of other works relating to general systematics theory but never directly cited in the text of any of the four volumes^1^ (Krammer & Lange‐Bertalot, 1991a). Interestingly, an early diatom contribution to cladistic taxonomy (Kociolek et al., 1989) was not included in the reference list. Whatever merits that can be found in Krammer and Lange‐Bertalot's interpretation of these various (and very different) contributions to the workings of systematics, they did at least appear to consider some of them, even if superficially and only for the species rank.

No such discussion is included in the revised versions of the *Süßwasserflora*, now entitled *Diatomeen im Süßwasser‐Benthos von Mitteleuropa* (Hofmann et al., 2011) and, with a more comprehensive title in its English translation, *Freshwater Benthic Diatoms of Central Europe over 800 Common Species used in Ecological Assessment* (Lange‐Bertalot et al., 2017, adapted from the title of the revised German edition, Hofmann et al., 2013; incredibly a supplement is now available, Werum et al., 2024). Instead, while acknowledging the influence of what is referred to as the “Era of the Green Books” (Krammer & Lange‐Bertalot, 1986, 1988, 1991a, 1991b, 2000a, 2000b, 2004, so called because the four earlier books that made up the *Süßwasserflora von Mitteleuropa*, as well as the reprinted versions, had green covers), Lange‐Bertalot et al. write that “These are exciting times for those interested in the phylogeny, biogeography and diversity of diatoms, but also hugely frustrating times for those concerned with the routine identification of diatoms for practical purposes” (p. 9). “Hugely frustrating times”? How so? How could “exciting times” for some be “hugely frustrating” for others when the same subject is being addressed?

I leave this for a fuller discussion another time, but for now, just consider that one reason for this frustrating dichotomy might be because “Application of the EU‐WFD [Water Framework Directive] needs a *consistent approach* to the naming of the organisms upon which assessments depend” (Lange‐Bertalot et al., 2017, p. 9). One assumes that “a consistent approach” means using the identifications in this book, and this book only—or, as some have suggested, even better than this book, “a [DNA] barcode system that largely emulates the widely used freshwater flora of Krammer & Lange‐Bertalot” (Mann et al., 2010, p. 568). Zimmermann et al. (2015) directly attach their “morphological method” to the WFD: “For the morphological analysis of the diatom composition in each sample … counting procedures were following standard procedures (European‐Committee‐for‐Standardization, 2003, 2004; Kelly et al., 1998), which correspond to the strategy of the WFD” (Zimmermann et al., 2015, p. 531).

After digesting the preamble to these recent floras, one might come away assuming that taxonomy has no theoretical basis worth discussing and is therefore not really a science but a technique, or set of techniques, that happens to be a flawed method, that it lacks relevant data, lacks constancy, and is almost entirely dependent on those who are “experts.” It is as if the natural world, that world we live in, is understood to be populated by some fixed number of species, and it is the job of the taxonomist to find them all, tell users what they are, and, please, find them quickly (why are you taking so long? “As for many other microorganisms, diatom taxonomy is still not resolved since *most taxa* remain undescribed,” Vasselon et al., 2025, p. 56; most taxa? How could you possibly know that?). Worse, it is as if taxonomy has no purpose other than to supply a series of names (binomials) for those more “inspired” disciplines such as ecological or paleoecological research programs, of which most, in Europe at least, are directed toward the WFD (Kelly et al., 2018).

Taxonomy, like science in general, requires a certain amount of devotion to collecting data—even if it is a “trade secret” that doing so is considered a pretty tedious task. Still, tedious or otherwise, it has to be done—little progress is made without at least some data collection, that *time‐consuming activity*. One might try and sidestep that activity by finding a way that works faster and more efficiently, eliminating the tedium, but in the end, one still has to have data—and not any data: *relevant data*. That requires some knowledge of how taxonomy works or is supposed to work (Williams & Ebach, 2008, 2020)—acquiring such knowledge might even solve the puzzle of why some remain “hugely frustrated” during these apparent “exciting times” (see Williams & Ebach, 2008, 2020). But simply collecting data is (almost) a pointless exercise. Data do not come readily explained. They require analysis and consideration, and the understanding that not all data are relevant, even some DNA data. Data collection might be tedious, but once collected, data require some intellectual input: You have to know what you are doing.

Perhaps, it is unwise to conclude with a lengthy quotation and one that focuses on cladistics, conceived here as an understanding of systematics rather than a methodology (see Williams & Ebach, 2008, 2020), but hopefully its significance should be clear:

If cladistics is merely a restatement of the principles of natural classification, why has cladistics been the subject of argument? I suspect that the argument is largely misplaced, and that the misplacement stems, as de Candolle suggests, from confounding the goals of artificial and natural systems. … More importantly, perhaps, because of the search for some ultimate artificial system (the ultimate source of data, or the ultimate clustering algorithm, that by itself would guarantee a worthwhile result, inaugurate a modern age of systematics, and in the process relieve us all of a heavy burden—reading the systematic literature of the past and reaching an informed judgement of its relevance). Beginning even before “the new systematics,” many different artificial systems (data sources and methods) were hailed as the salvation of systematics; all ultimately have failed, if only because no artificial system can prevail as natural; it is nature that prevails. (Nelson, 1979, p. 20)

“II faloit chercher dans la nature elle‐méme son Systeme, s'il etoit vrai qu'ele en eut en” (Adanson, 1763, p. clvii). For all his idiosyncratic views, Michel Adanson (1763–1764, see Lawrence, 1963–1964) braved the stormy waters of biological classification and attempted to achieve something natural, something in nature itself (Williams & Ebach, 2020; Winsor, 1994). From my perspective, he failed. However, he fought his own battles, and in spite of defeat, others have fought for his ideals, if not his version, under another name (e.g., cladistics, Nelson & Platnick, 1981; Williams & Ebach, 2020). Regardless of motivation, nature will indeed prevail.

## AUTHOR CONTRIBUTIONS

**David M. Williams:** Conceptualization (equal); investigation (equal); writing – original draft (equal); writing – review and editing (equal).

## Footnotes

^1^
The 5th volume (Krammer & Lange‐Bertalot, 2000a), a series of keys for use with the previous four volumes, the text in English and French, included a few additional references.
